# A rare case of schizophrenia coexistence with antiphospholipid syndrome, β-thalassemia, and monoclonal gammopathy of undetermined significance

**DOI:** 10.3389/fpsyt.2023.1178247

**Published:** 2023-04-06

**Authors:** Yingming Jin, Yiquan Cheng, Jifeng Mi, Jianfen Xu

**Affiliations:** ^1^Department of Hematology and Oncology, Ningbo No.2 Hospital, Ningbo, China; ^2^Department of Laboratory Medicine, Ningbo No.2 Hospital, Ningbo, China

**Keywords:** β-thalassemia, antiphospholipid syndrome, chlorpromazine, lupus anticoagulant, MGUS, schizophrenia

## Abstract

A patient with schizophrenia who was treated with chlorpromazine developed lupus anticoagulant (LA) and antiphospholipid syndrome (APS). On protein electrophoresis, a monoclonal immunoglobulin A peak was seen in this patient, defining a condition of monoclonal gammopathy of undetermined significance. Additionally, β-thalassemia was diagnosed with the CD41-42 genotype. This condition is extremely rare, particularly in patients with schizophrenia and APS. We present a case of a patient with schizophrenia and secondary APS who had a positive LA, a significantly prolonged activated partial thromboplastin time, endogenous coagulation factor deficiency and inhibitor, no bleeding, and an unexpected finding of β-thalassemia and monoclonal IgA. Following that, a literature review on the disorders was presented.

## Introduction

Schizophrenia, which affects up to 1% of the population, is recognized as one of the most serious, disabling, and costly psychiatric disorders (1). It is characterized by delusions, hallucinations, cognitive impairment, social withdrawal and impaired memory (2, 3). For decades, hypotheses of dopaminergic and glutaminergic systems are involved in the pathophysiology of schizophrenia (3, 4). Studies have showed lower gray matter volume in patients with schizophrenia and higher rates of gray-matter loss in prefrontal and parahippocampus areas in people with prodromal psychosis than healthy persons (4, 5). Successful identification of numerous susceptibility loci has provided useful insights into the molecular etiology of schizophrenia (6–8). Recently, the immunological hypothesis has gained prominence and autoimmune diseases are thought to be causally related to schizophrenia (9–12). However, there is no central pathophysiology mechanism have been defined for schizophrenia.

Antipsychotic drugs are mainly used for treating schizophrenia and significantly improve the outcomes of treatment at all stages of the disease (3, 13). Early treatment plays an important role in schizophrenia and can help improve long-term outcomes of schizophrenia (14, 15). However, antipsychotic medications usually attenuate positive symptoms, but fail to improve negative features or cognitive function and they also come with some serious potential side effects (16). The common side effects of antipsychotic medications are weight gain, sexual side effects, and extrapyramidal effects while, other rare side effects of antipsychotic medications that have received less attention include dysfunction of blood coagulation and autoimmune disorders. Patients who experience side effects are more likely to affect their adherence to prescribed medical therapy and give up medication (17).

Antiphospholipid syndrome (APS) is an autoimmune disorder characterized by antiphospholipid (aPL) antibodies, arterial and venous thrombosis, pregnancy morbidity, and various neurological manifestations, including psychiatric disorders. The majority of APS pathogenic mechanisms are complex or unknown (18). Comparisons of schizophrenia patients and healthy subjects have revealed differences in immunologic parameters (19). There has been repeated evidence that a genetic locus for schizophrenia in the region of the human leukocyte antigens (HLA) (20). In addition, a prior autoimmune disease increased the risk by 29% (10). Several studies have suggested that antibodies to aPL are associated with schizophrenia (21–24).

β-thalassemia is one of the world’s most common genetic diseases, caused by mutations in the beta chain of the hemoglobin molecule (25). Although not life threatening on its own, the effects of mild-to-moderate anemia can have an impact on quality of life. In certain Mediterranean families, very rare cases of β-thalassemia coexisting with psychiatric disorders have been reported (26).

Monoclonal gammopathy of undetermined significance (MGUS) is characterized by the presence of a monoclonal paraprotein in the blood, without the characteristic end organ damage seen in multiple myeloma. Numerous studies have found an increased incidence of MGUS in patients with autoimmune diseases, and their treatment may play an important role in the etiology of MGUS (27, 28). However, the presence of monoclonal immunoglobulin and APS in the same patient is unusual.

The coexistence of four diseases in a single person deserves our attention, particularly given the growing interest and research on this topic. We first described a rare case of schizophrenia accompanied by APS, β-thalassemia, and MGUS in this paper. Additionally, in this paper, we described a rare side reaction during treatment, chlorpromazine-induced LA, prolonged APTT and APS, which makes more difficults in our clinical treatment. Then, we presented a comprehensive literature review on the disorders.

## Case presentation

A 64-year-old man who was born in Zhejiang Province, went to consult doctor at the age of 30 for auditory hallucinations and impulsive communication with others for about 2 years. The patient was eventually diagnosed of schizophrenia with predominantly positive symptoms. He was initially treated with chlorpromazine at a low dose and later with 250 mg/day. After 1 months of treatment, he reported decrease in frequency of hallucinations. However, about 1 or 2 years later, the patient discontinued to take the medications according to his own will. Unfortunately, his symptoms got worse again. After a series of examination, his attending physician diagnosed the patient with a relapse. Therefore, he had to get treatment again with a dose of 250 mg/day without any visible traces of blood. Although he was irritable and spoke abusively, His paranoid delusion improved and he restored social functions. On 19 November 2021, he was admitted to our hospital with anemia and a significantly prolonged activated partial thromboplastin time (APTT) without bleeding. His previous medical history included anemia for 30 years without further diagnosis or treatment, because of left lower limb deep venous thrombosis and pulmonary embolism, an inferior vena cava filter was inserted on 30 May 2013, and warfarin was administered for about 1 year. Two episodes of transitory ischemic attack occurred in 2021, and colonoscopy revealed multiple intestinal polyps, which were removed on 1 September 2021. Laboratory evaluation revealed mild anemia [hemoglobin of 90 g/L, mean corpuscular hemoglobin concentration of 311 g/L, mean corpuscular volume of 61.7 fl, and mean corpuscular hemoglobin of 19.2 pg, with normal white blood cells (9.3 × 10^9^/L) and platelet (315 × 10^9^/L)]. Coagulative studies revealed that prothrombin time (PT) was 17.5 s, APTT was 80.7 s, fibrinogen was 755 mg/dL, and D-dimer was 174.0 ng/mL (Table 1).

**TABLE 1:** Results of routine blood count and coagulation tests with abnormal test results.

	Results	Reference intervals
**Routine blood count**		
Leukocytes, 10^9^/L	9.3	3.50–9.50
Neutrophils, %	79.7	40–75
Lymphocytes, %	11	20–50
Hemoglobin, g/L	90	130–175
Mean corpuscular volume, fl	61.7	82–100
Mean corpuscular hemoglobin concentration, g/L	311	316−354
Mean corpuscular hemoglobin, pg	19.2	27.0−34.0
Platelets, 10^9^/L	315	125–350
**Routine coagulation tests**		
PT, seconds	17.5	9.4−12.5
aPTT, seconds	80.7	25.1−36.5
INR	1.51	0.88−1.25
Fibrinogen, mg/dL	755	276−471
D-dimer, ng/mL	174	<243

After reviewing the history, the patient was admitted to the hospital in 2013 for pulmonary embolism (PE) with a significantly prolonged APTT of 68 s, which was not taken seriously. A mixing study with normal plasma 1:1 (29, 30) revealed no APTT correction (Table 2).

**TABLE 2:** APTT mix test and LA test.

	Results	Reference range
Rosner index	57 s	
APTT (normal plasma)	28.8 s	26−43 s
APTT (normal plasma 2 h incubation)	29.1 s	
APTT (the patient’s plasma)	80.7 s	26−43 s
APTT (the patient’s plasma 2 h incubation)	89.5 s	
**Mix studies show below**		
**APTT patient plasma: normal pooled plasma (1:1)**		
Immediate result	74.7 s	
2 h incubation result	74.6 s	
dRVVT screen ratio (LA1)	2.17	
dRVVT confirm ratio (LA2)	1.00	
dRVVT normalized ratio (LA1/LA2)	2.17	0.5−1.2
R0-silica clotting time (SCT)-S	2.56	
R0-SCT-C	1.14	
SCT normalized ratio	2.25	0−1.2

Subsequently, a lupus anticoagulant (LA) test and coagulation factors were performed (Tables 2, 3 and Supplementary Table 1). LA activity was considered strongly positive by the dilute Russell viper venom Time (dRVVT) test (31) (screen-to-confirm ratio, 2.17; normal range 0.5−1.2). Accordingly, we confirmed that the patient’s prolonged APTT was caused by LA interference. The antinuclear antibody (ANA) level was 1:80 (homogeneous type). The anticardiolipin (aCL) antibody was 141 PLU/mL, and the anti-β2 glycoprotein I (anti-β2GPI) antibody was 47 RU/mL.

**TABLE 3:** Result of coagulation factor and antiphospholipid antibody.

	Result	Reference value
**Coagulation test**		
Factor II activity	94%	70−120
Factor V activity	95%	70−120
Factor VII activity	62%	55−170
Factor VIII activity	2%	70−150
Factor IX activity	5%	60−150
Factor X activity	81%	70−120
Factor XII activity	9%	60−150
Factor VIII antibody	2.2BU	<0.6BU
Anti-thrombin III	103%	80−120
Protein C functional	97%	70−130
Protein S functional	152.2%	55−140
**Antiphospholipid antibody and antinuclear antibody**		
Antinuclear antibody	1:80	Negative
aCL antibody	141 PLU/ml	<12 PLU/ml
Anti-β2GPI antibody	47 RU/ml	<20 RU/ml
Antiphosphatidylserine antibody	40 RU/ml	<20 RU/ml

**FIGURE 1:**
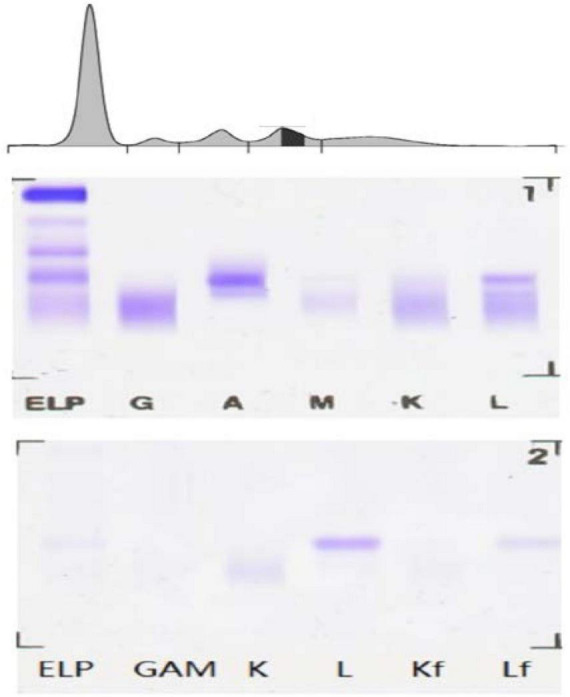
Serum electrophoresis and immunofixation electrophoresis of the patient.

Proteins C and S and antithrombin III levels were within normal range. The reticulocyte count was 0.051 × 10^12^/L, and the iron metabolism was as follows: serum iron of 3.4 μmol/L, serum ferritin of 196 ng/mL, and total iron-binding capacity of 41.4 μmol/L. Erythropoietin, folic acid, vitamin B12, and β_2_ microglobulin levels were normal. The Coombs test was negative. The level of immunoglobulin A was 5.97 g/L, and immunofixation electrophoresis (IFE) revealed IgA-λ (Figure 1).

A bone marrow biopsy revealed normal marrow with a 1% plasma cell population. The erythroid hyperplasia was clearly active, with no internal or external iron deficiency. Immunotyping revealed that 0.5% abnormal cells expressed CD27, CD38 (bright), CD138, and cLambda but not CD19, CD28, CD56, and cKappa. His karyotype was normal, and he had the β-thalassemia mutation (genotype of CD41–42) (Supplementary Figure 1). Vascular ultrasound indicated venous thrombosis in the left upper extremity, and pulmonary artery CTA revealed no evidence of pulmonary embolism. Finally, a diagnosis of schizophrenia, CPZ-induced LA and antiphospholipid syndrome, β-thalassemia minor (CD41–42 heterozygous mutation), and MGUS was made. Therefore, the patient stopped taking CPZ and switched to perospirone initially with a low dose of 4 mg, later with 16 mg per day. The mental state of our patient was stable and there were no report of discomfort. The potential bleeding risk must be carefully considered due to the adverse effects of prolonged anticoagulation combined with prolonged APTT. Furthermore, the patient’s adherence to anticoagulant therapy was poor. Therefore, the patient did not receive anticoagulant therapy at first. The prolonged APTT gradually improved, decreasing from 80.7 to 44.4 s (Supplementary Table 2). Unfortunately, he had recurrent left lower limb deep venous thrombosis by ultrasonography on 18 September 2022. Subsequently, an inferior vena cava filter was inserted. He had to received anticoagulant therapy with rivaroxaban (initially 15 mg by mouth twice daily 3 weeks, followed by 20 mg per day for maintenance) without any follow-up of coagulation function or thrombus for personal reasons.

## Discussion

Antiphospholipid syndrome is a prothrombotic disease characterized by persistently elevated levels of aPL antibodies and clinical manifestations of venous or arterial thrombosis. LA, as a member of the aPL antibodies, is a polyclonal immunoglobulin that interferes with the *in vitro* phospholipid-dependent coagulation assay. Typically, this interference does not result in PT extension, whereas APTT prolongation is determined by the agentic used for APTT detection (32). Moreover, bleeding manifestations are uncommon in LA-positive patients (33). Notably, we found low levels of coagulation factors VIII (FVIII), IX, and XI, as well as VIII antibody. It is uncommon in the literature to find LA combined with endogenous coagulation factor deficiency and inhibitor (34, 35). Actually, LA can interfere with the detection of endogenous coagulation factor activity, causing false results, and the dilution assay can be used to obtain more accurate coagulation factor levels by lowering the titer of interfering substances (36). Hence, this patient may be affected by LA as well, resulting in a false reduction of endogenous coagulation factors. Unfortunately, no dilution test was performed because the coagulation factor measurement was performed in an external laboratory with limited detection capacity, and the patient refused to re-examine for financial reasons. Therefore, we were unable to evaluate this in our case.

Schizophrenia is a serious chronic mental illness that affects approximately 1% of the population. Antipsychotic drugs are the primary treatment with schizophrenia. CPZ, blocks dopamine receptors, is one of the most commonly used first-generation drug treatments for people with schizophrenia, whereas perospirone is a second-generation antipsychotic that is a partial serotonin (5-HT)1A receptor agonist, as well as a 5-HT2A, D2 dopamine, and α−1 adrenergic receptor antagonist (4, 37). Previous research has shown that aPL antibodies can appear as a result of antipsychotic drug treatment. The prevalence of LA and aCL antibodies in neuroleptic treated users was 24.4 and 21.7%, respectively, in the largest published study of 184 patients with chronic psychosis (38). A series of observations revealed the prevalence of aPL antibodies in CPZ patients increased when compared with other antipsychotics (22, 39, 40). Ducloux et al. (41) reported a 25-year-old woman that had received CPZ for 3 years when she was taken to the hospital with inferior vena cava thrombosis and PE. Subsequent assays showed increased aCL antibodies which falled to the normal range following the replacement of other drugs. Recently, Regina et al. (24) described 5 patients with schizophrenia out of 150 patients with APS. However, similar reports are rarely documented. Although a coagulation pathway abnormality and immune-related phenomena have been proposed as links between aPL antibodies and schizophrenia, coexistence of schizophrenia in patients with definite APS is uncommon, and the mechanisms of this association remain unknown (24, 42, 43). Interestingly, to the best of our knowledge, no reports of perospirone inducing aPL antibodies or abnormal coagulation function have been found. Given the use of antiepileptic medications, we believe that CPZ-induced LA and aCL antibodies are more likely. However, we cannot ignore the fact that APS can manifest as central nervous system psychosis and schizophrenia may be a complication of APS (44). Additionally, unmedicated mental patients and their healthy relatives had a high incidence of aPL antibodies, and there was no difference in aPL antibody levels between treated and untreated schizophrenia patients (45). After discontinuing CPZ and switching to perospirone, the patient’s prolonged APTT showed a moderate decrease from 80 to 44.4 s. Therefore, we prefer to focus on the diagnosis of CPZ-induced LA and APS.

β-thalassemia is a group of genetic abnormalities in humans that range from asymptomatic to severe anemia. Southern China has a high incidence of the disease, and the most common mutation for β-thalassemia is CD41/42 (46–48). There have been case reports of β-thalassemia coexisting with psychiatric diseases in some Mediterranean families. However, according to our literature review, only one report showed that two family members were diagnosed with schizophrenia and β-thalassemia minor (heterozygous β-thalassemia), implying that the two diseases may be linked genetically (26). Several studies have found a possible genetic susceptibility to schizophrenia on the short arm of chromosome 11, near the gene involved in β-thalassemia (49). β-globin genes are found at 11p15.5, near tyrosine hydroxylase, dopamine receptor D4, and brain-derived neurotrophic factor, which are related to mental disorders (50). Moreover, one of the human tryptophan hydroxylase genes, which has been linked to schizophrenia, is found on the short arm of chromosome 11 (11p14–p15.3) (51). We believe that future research on hemoglobin will pave the way for the discovery of new pathogenesis of psychiatric disorders.

It is unusual for a patient to have LA and monoclonal gamma globulin disease. The mechanism of aPL antibody development is still unknown. Because APS and monoclonal gamma globulin disease increase the risk of thrombosis, thrombotic events may be caused by the combined effect of these conditions (52). Recently, Doyle et al. (53) described a new discovery of a small number of patients with thrombotic APS and monoclonal gamma globulin receiving anticoagulation therapy having a higher incidence of recurrent thrombosis than patients without accessory protein. When thrombotic APS develops, long-term anticoagulation is required. Vitamin K antagonists are preferred anticoagulants, particularly when aPL antibodies are serologically triple-positive (54). Khamashta et al. (55) reported a thrombosis recurrence rate of 20 to 40% per year without anticoagulant treatment. Ten months after discontinuing anticoagulant therapy, the patient developed recurrent venous thrombosis. However, there were no standardized treatment regimens or guidelines for APS-associated psychosis. Warfarin is a recommended anticoagulant in APS patients with triple-positive aPL antibodies (56). Additionally, Hoirisch-Clapauch et al. (57) demonstrated that warfarin alone can improve the symptoms of psychosis in APS patients. However, warfarin therapy presented a number of challenges. In the midst of the recent COVID-19 outbreak, it is difficult for patients with schizophrenia to monitor their coagulation function and INR while on warfarin therapy, let alone visitor, and consult their clinicians. Hence, the patient was prescribed rivaroxaban to treat APS with thrombosis recurrence.

The limitations of our case are as follows: due to the patient’s special condition, poor compliance, and his poor financial reasons, the changes of antiphospholipid antibodies and coagulation factors in the patient’s body could not be evaluated after drug adjustment. Not to mention genetic testing to further clarify the link between schizophrenia and thalassemia. Also, the dilution of coagulation factor test could not be performed in an external laboratory with limited detection capacity.

We first described a rare case of schizophrenia with concurrent APS, β-thalassemia, and MGUS, as well as a markedly prolonged APTT, without bleeding symptoms. Given the condition of the patient in our case, we speculate that CPZ induced LA and APS. However, more research is needed to uncover the complex biology of these disorders to improve clinical management of these patients. Despite the fact that current knowledge is limited, we believe clinicians, particularly psychiatrists, should be aware of this possible adverse drug reaction and consider interrupting or switching treatment in patients who exhibit this reaction. In our case, its significance lies in CPZ-induced LA and APS which is easily overlooked by psychiatrists. Furthermore, we emphasize that when a patient has APS, a prolonged APTT, and a lack of coagulation factors, clinicians must carefully weigh the risks of thrombotic events versus bleeding. More research is needed to better understand the complex biological mechanisms.

## Data availability statement

The original contributions presented in this study are included in the article/Supplementary material, further inquiries can be directed to the corresponding author.

## Ethics statement

The studies involving human participants were reviewed and approved by the Ningbo No.2 Hospital. The patients/participants provided their written informed consent to participate in this study. Written informed consent was obtained from the individual(s) for the publication of any potentially identifiable images or data included in this article.

## Author contributions

YJ reviewed the relevant literature and wrote the draft manuscript. YC verified the relevant content and revised the manuscript. JM provided the relevant original images. JX created, reviewed, and revised the work. All authors contributed to manuscript and agreed to submit this manuscript.
