# Supplementary material for: A rare case of schizophrenia coexistence with antiphospholipid syndrome, β-thalassemia, and monoclonal gammopathy of undetermined significance
Source: Front Psychiatry. 2023 Apr 6;14:1178247. doi: 10.3389/fpsyt.2023.1178247 (PMC10117972; doi:10.3389/fpsyt.2023.1178247)
Supplement: Supplementary file 1 [file Data_Sheet_1.zip › supplementary material.pptx]

## Slide 1
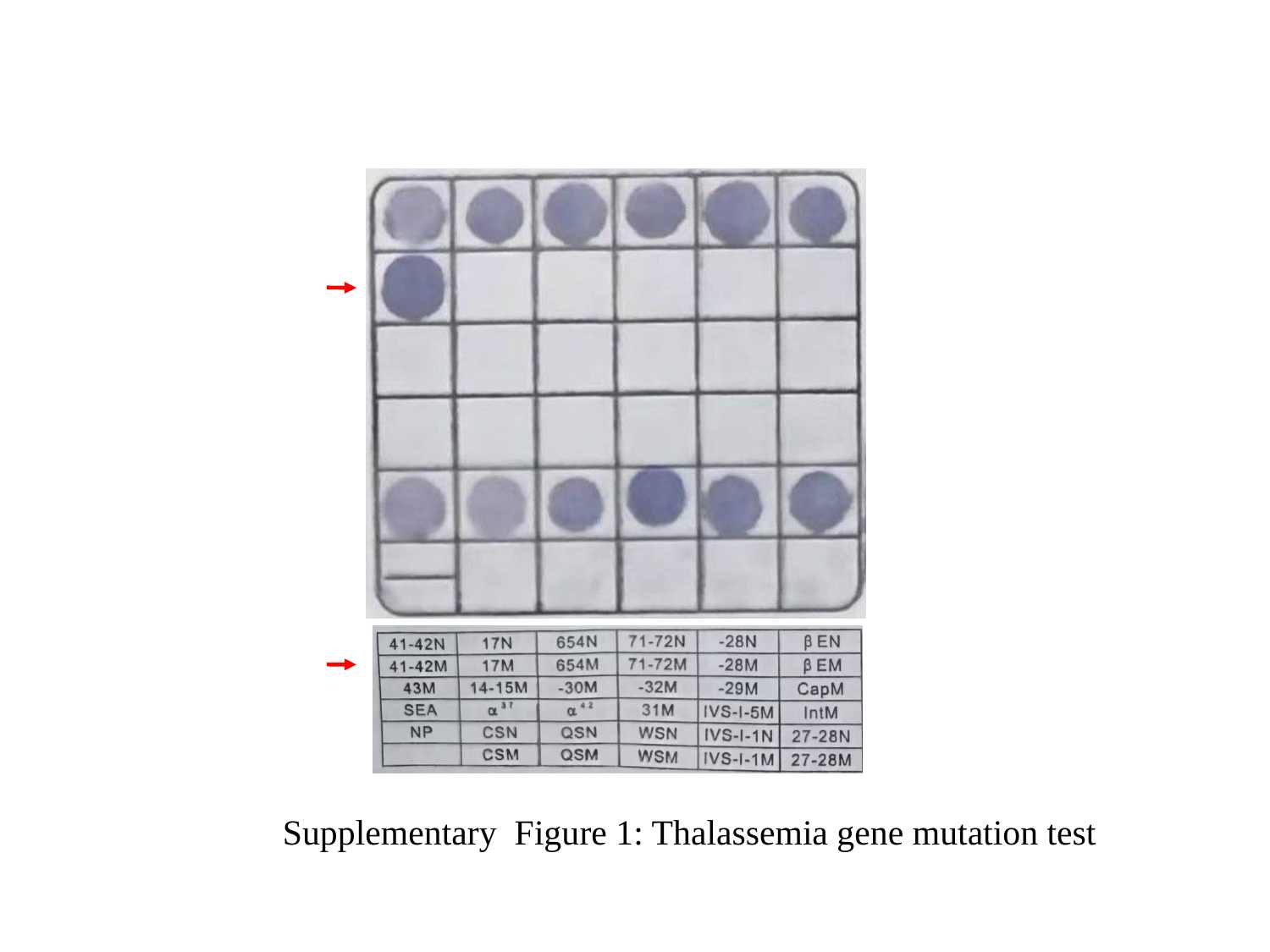

Supplementary Figure 1: Thalassemia gene mutation test

## Slide 2
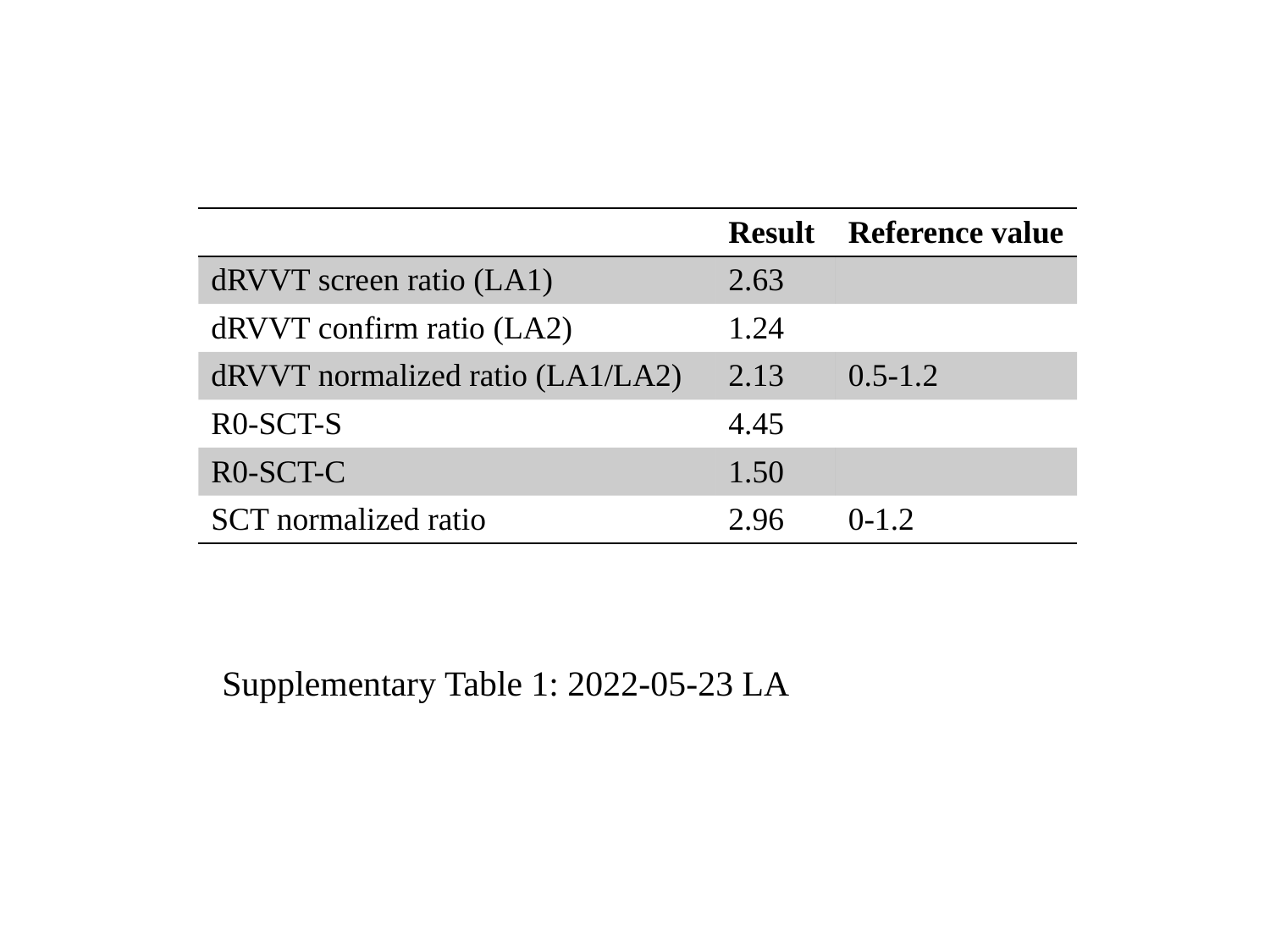

| | Result | Reference value |
| --- | --- | --- |
| dRVVT screen ratio (LA1) | 2.63 | |
| dRVVT confirm ratio (LA2) | 1.24 | |
| dRVVT normalized ratio (LA1/LA2) | 2.13 | 0.5-1.2 |
| R0-SCT-S | 4.45 | |
| R0-SCT-C | 1.50 | |
| SCT normalized ratio | 2.96 | 0-1.2 |
Supplementary Table 1: 2022-05-23 LA

## Slide 3
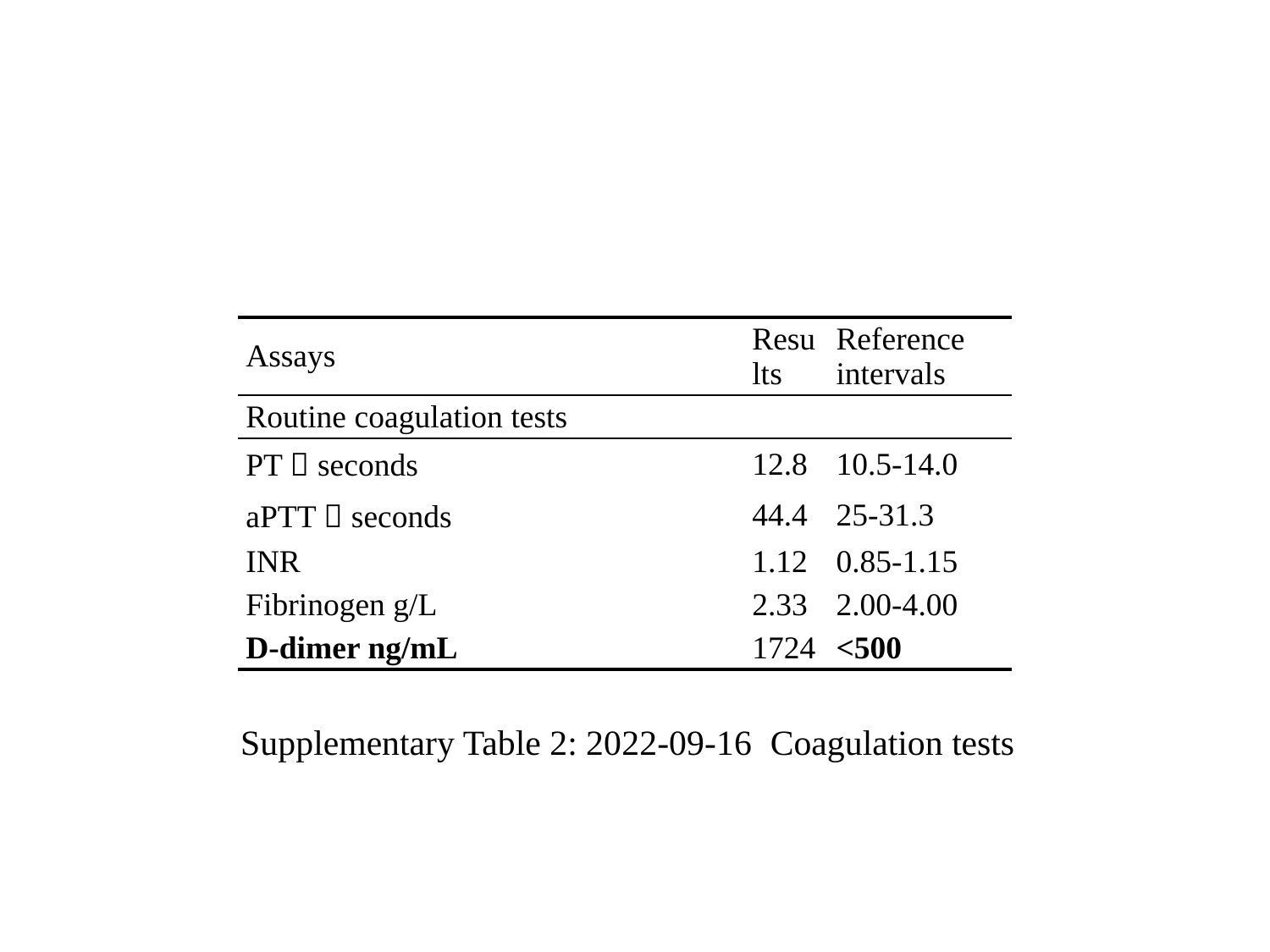

| Assays | Results | Reference intervals |
| --- | --- | --- |
| Routine coagulation tests | | |
| PT，seconds | 12.8 | 10.5-14.0 |
| aPTT，seconds | 44.4 | 25-31.3 |
| INR | 1.12 | 0.85-1.15 |
| Fibrinogen g/L | 2.33 | 2.00-4.00 |
| D-dimer ng/mL | 1724 | <500 |
Supplementary Table 2: 2022-09-16 Coagulation tests
